# Work-related musculoskeletal disorder among midwives, a threat for maternity care

**DOI:** 10.1371/journal.pone.0339962

**Published:** 2025-12-31

**Authors:** Osman Yimer Mohammed, Kerstin Erlandsson, Tewodros Seyoum, Solomon Hailemeskel, Lemma Derseh, Helena Lindgren

**Affiliations:** 1 Department of Women and Family Health, School of Midwifery, College of Medicine and Health Sciences, University of Gondar, Gondar, Ethiopia; 2 Midwifery Department, School of Nursing and Midwifery, Asrat Woldeyes Health Science Campus, Debre Berhan University, Debre Berhan, Ethiopia; 3 School of Health and Welfare, Dalarna University, Falun, Sweden; 4 Department of Women’s and Children’s Health, Karolinska Institutet, Stockholm, Sweden; 5 Department of Epidemiology and Biostatistics, Institute of Public Health, College of Medicine and Health Sciences, University of Gondar, Gondar, Ethiopia; 6 Department of Health Promotion, Sophiahemmet University, Stockholm, Sweden; Bangladesh University of Engineering and Technology, BANGLADESH

## Abstract

**Background:**

Work-related musculoskeletal disorder is a limiting, painful condition that affects the muscular, skeletal, articular, and nervous tissues of the body. The condition is mainly associated with poor working conditions and awkward body positions. Health professionals, including midwives, are among the most affected workforce globally**.** The condition affects the health of the professionals and the quality of care that professionals are expected to provide. However, there is a scarcity of information on the magnitude of the condition, its effect on midwifery practice, and associated factors. Objectives: This study aimed to assess the magnitude of work-related musculoskeletal disorder, its effect on midwifery practice, and associated factors among midwives in North Shoa Zone, Amhara Regional State, Ethiopia.

**Method:**

An institution-based cross-sectional study assessed the magnitude of work-related musculoskeletal disorder (WRMSD), the effect on midwifery practice, and associated factors. The Nordic Musculoskeletal Disorder Scale was used to assess the presence of WRMSDs in the nine regional body parts and its effect. A stepwise backward elimination logistic regression model was used, and significant association was declared at a p-value of less than 0.05.

**Results:**

A total of 473 (252 (53.3%) female and 221 (46.7%) male) midwives participated in this study. Overall, in the last 12 months, 355 (75.05%, 95% CI: 71.15% − 78.95%) midwives were affected by WRMSD, which was seen in any one of the nine regional body parts. About 45% (162) of them reported being unable to perform their daily tasks while they were affected, and 27% (96) of them sought treatment for their condition. The lower back was the most affected axial body part, reported by 300 (63.4%, 95% CI: 59% − 68%) midwives. Working in awkward or uncomfortable positions was significantly associated with the development of WRMSD (AOR: 1.81, 95% CI: 1.15–2.87). Similarly, awkward positions significantly affected the development of area-specific WRMSD in the lower back, upper back, neck, and limbs. Working in the same position for a longer time, seeing clients daily, and moving heavy objects were among the risk factors associated with developing area-specific WRMSD among midwives.

**Conclusion:**

The magnitude of work-related musculoskeletal disorders is high among midwives, and a significant number of them were unable to perform their daily tasks. The lower and upper back are the most commonly affected areas. Working in uncomfortable positions and attending to large clients daily were common risk factors. Therefore, training midwives about safe working positions and reducing the workload is commendable.

## Introduction

Musculoskeletal disorder (MSD) is a health condition that affects the locomotion system, including muscles, tendons, the skeleton, cartilage, ligaments, and nerves. WRMSD is caused or intensified by working conditions [1]. The condition is referred to as work-related musculoskeletal disorder (WRMSD) when it is caused or intensified by working conditions [2]. The disorder occurs when the workload exceeds the capacity of the body parts and causes injuries. These injuries include strain and rupture of the muscle and tendon, microfractures, and fractures of the bones [1,3].

The condition develops with no symptoms until it causes significant damage and pain; the process may take months to several years [3]. Musculoskeletal injuries first occur due to overstretching, compression, friction, ischemia, or overextension, which leads to inflammatory responses. The cyclical nature of exposures to workplace risks contributes to the repetition of injuries and inflammatory responses. This process leads to the development of fibrotic tissue and tissue damage, which eventually causes pain and loss of motor function [2,4]. The condition occurs as both acute and chronic conditions. Acute WRMSD occurs when a heavy load damages the muscles or breaks the bones. Chronic conditions arise when an overload of work causes increasing pain due to wear and tear of the musculoskeletal structures [1].

The risk factors for the development of WRMSD can have individual, physical, or psychosocial characteristics [5]. Individual-related risk factors include being middle-aged or older, being female, level of educational qualification, having training in safety, work experience, having a high body mass index (BMI), and comorbidity [6–9]. ork-related causes include overload, work that requires repetitive movement, heavy physical work, the need for excessive force, the need to be in awkward and/or sustained postures, prolonged time in a similar position, longer shifts, fewer breaks, having high psychosocial work demands, job stress, lack of support, and the nature of the work unit [1,4,6,10–13].

Health professionals are among the most affected workers globally [6]. Midwives’ working postures cause unnatural body alignments, non-ergonomic spinal alignment, and postural instability [14]. This makes midwives one of the most commonly affected health professionals by WRMSD [9,15–18]. The prevalence has been reported from 40% to 90% [9,19,20]. The most commonly affected body parts are the lower back, neck, shoulder, elbow, and limbs [7,8,20,21].

WRMSD, being a health problem that causes suffering and pain, is associated with job burnout and job stress. These conditions are associated with limited service efficiency, low quality of care, and eventually leaving the profession. WRMSD incurs direct costs on workers and employing companies for healthcare, and indirect costs through a reduction in employee productivity [3,8] However, there is no adequate evidence about the magnitude of the condition globally in general and in the Sub-Saharan region specifically. Additionally, midwives are exposed to high work load and the work demands non ergonomic body position [14,22]. Therefore, this study aimed to investigate the magnitude of work-related musculoskeletal disorder and associated factors among midwives in North Shoa Zone, Amhara Regional State, Ethiopia.

## Materials and methods

### Study design

We conducted an institution-based cross-sectional study from October to November 2024 in North Shoa Zone, Amhara Regional State, Ethiopia, among midwives working in public health facilities. North Shoa Zone, one of the eleven zones in the region, has 24 Woredas and three city administrations. The zone’s capital, Debre Berhan, is located 130 km from the capital, Addis Ababa. A total of eleven hospitals (one referral hospital, two general hospitals, and eight primary hospitals) and 91 health centers provide healthcare services in the zone.

### Study population

The study population included all midwives working in the health facilities found in North Shoa Zone, Amhara National Regional State, Ethiopia.

#### Inclusion criteria.

Midwives who were working in the public health facilities in the study area, had more than one year of work experience, and participate in maternity healthcare services were included.

#### Exclusion criteria.

Midwives who were working in private health facilities, critically ill midwives who were unable to communicate, student midwives, and midwives on maternity or sick leave were excluded.

### Sample size and sampling procedure

A census of all the midwives working in public health facilities (473) who were available during data collection and fulfilled the inclusion criteria was conducted. A census, a complete inclusion of all study populations, approach was preferred to obtain accurate data and the whole image of the population since the study was conducted in a limited study area and the number of midwives in the study area was not too much [23].

### Variables and measurement

#### Outcome variable.

Work-related musculoskeletal disorder (WRMSD).

#### Independent variables.

Age, sex, level of education, facility type, marital status, family size, height, weight, working position, and workload.

### Data collection process

Data were collected using an interviewer-guided self-administered questionnaire. WRMSD was assessed using the Nordic Musculoskeletal Questionnaire (NMQ), a tool that was developed and validated for the analysis of musculoskeletal symptoms in nine regions of the body [24]. Accordingly, the presence and absence of WRMSD in nine parts of the body (neck, shoulder, elbow, wrist/hand, upper back, lower back, hip/thigh, knee, and ankle/feet) over the past 12 months and 7 days, respectively, were assessed. Midwives were asked to report if they had pain or discomfort in one of the nine regions irrespective of the severity or of effect of the pain on their daily activity. Accordingly, midwives who reported any pain were considered to have been affected by the overall last 12 months of WRMSD. Additionally, the prevalence of WRMSD for the nine body parts in the previous 12 months and the last seven days was estimated. Socio-demographic variables, academic level, and nature of the working environment were assessed to evaluate potential risk factors that affect the development of WRMSD.

### Data quality control

Training was provided to six BSc data collectors and two MSc supervisor midwives on the data collection tool over the course of one day. A pilot test was conducted on 5% of the study participants outside the study area. The supervisors guided the data collectors and checked the completeness of the data daily.

### Data analysis

The data were entered into Epidata version 4.6 and exported to STATA 17 for statistical analysis. Summary statistics (Mean, SD, Proportion, and Ranges) were used to summarize the data. Pictorial presentations, such as tables and graphs, were used to present the findings. A stepwise backward elimination logistic regression model was used to identify factors associated with WRMSD. All variables were included to the model first. Variables with highest p-value were removed while comparing the AIC and BIC of each model. The procedure was repeated until all the variables that causes the AIC and BIC to increase were removed. Eventually, the models with the smallest AIC and BIC were selected. The backward elimination was used to build a good fit model for prediction of WRMSD among midwives. Multicollinearity of the predictor variables and the model fitness were assessed using variable inflation factor (VIF, < 10 cut of point), and Hosmer and Lem show goodness-of-fit test (P-value >0.05), respectively. Statistical significance was declared at a p-value of less than 0.05.

### Ethical approval

This study was approved by the Ethical Committee of the College of Medicine and Health Sciences Specialized Referral Hospital (CMHSSH) under the University of Gondar Institutional Research Ethics Review Committee (IRERC), (CMHSSH-IRERC/14/11/2024). No minors were involved in the study, and a written informed consent was obtained from each study participant after providing detailed information about the purpose of the study. The anonymity of the data was maintained, and no personal identifying information was used during analysis and reporting.

## Results

This study recruited a total of 473 participants, comprising 252 (53.28%) female and 221 (46.72%) male midwives. A total of 17 midwives (three declined to participate, nine were not available at the time of data collection, two were on maternity leave, and three had incomplete records) were not included, yielding the response rate to be 96.53%. The mean age of the participants was 29.43 ± 3.8, ranging from 22 to 48 years. Most midwives, 292 (61.73%), were aged between 26 and 30 years. More than half, 251 (53.1%), of the midwives had a bachelor’s degree in midwifery. The body weight of 376 (79.5%) of the midwives was in the normal weight range (Table 1).

**Table 1:** Socio-demographic characteristics of the midwives who responded to the survey, North Shoa Zone, Amhara Regional State, Ethiopia, 2025 (n = 473).

Variable	Category	Frequency	Percentage
Sex	Male	221	46.72
	Female	252	53.27
Age	≤25	53	11.20
	26-30	292	61.73
	≥31	128	27.06
Ethnicity	Amhara	469	99.15
	Oromo	3	0.006
	Other	1	0.002
Religion	Orthodox	412	87.10
	Muslim	53	11.20
	Protestant	8	0.016
Marital status	Single	170	35.94
	Married	300	63.42
	Widowed & Divorced	3	0.006
Family size	1-2	416	87.94
	3-4	57	12.05
Academic level	Diploma	178	37.63
	BSc	251	53.06
	MSc	44	9.30
BMI	Underweight	35	7.39
	Normal weight	376	79.49
	Overweight and obese	62	13.11

### Work-related characteristics

**Fig 1:**
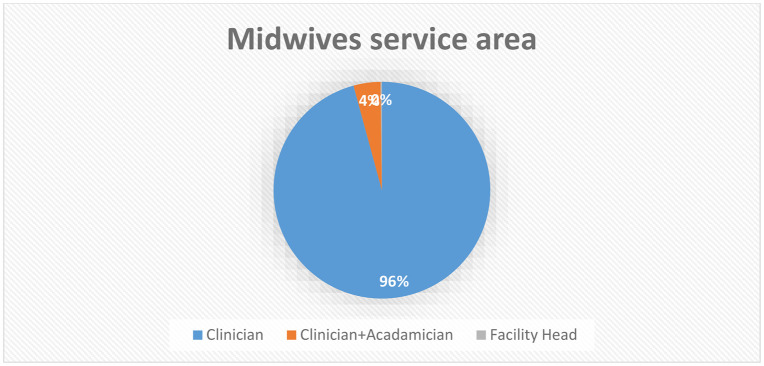
The proportion of midwives’ service area in the health sector, North Shoa Zone, Amhara Regional State, Ethiopia, 2025.

Most midwives, 453 (95.77%), served as clinical service providers, and only one midwife reported being a facility head. Most midwives, 451 (95.34%), were permanent employees; more than half of the midwives, 271 (57.29%), worked in health centers, and more than half, 254 (53.69%), of the midwives had work experience of 6–10 years. Most of the midwives, 402 (84.98%), participated in night duty, and more than half, 254 (53.69%), of the midwives had more than three night duties per week (Fig 1).

Midwives worked in the delivery, antenatal care (ANC), gynecology, and family planning units; they were assigned to different working units on a rotational basis, and most midwives worked in more than one unit. The labor ward was the central working unit for midwives, where 380 (80.33%) of them delivered their services, and about 215 (45.45%) midwives also worked in the ANC unit.

Regarding working conditions, most midwives, 373 (78.85%), reported having access to adjustable delivery couches. About 322 (68.07%) of the midwives reported that their work involved moving heavy objects, 350 (73.99%) of the midwives reported that they worked in the same position for a longer time, and 318 (67.23%) of the midwives reported that their work required them to assume awkward positions (Table 2).

**Table 2:** Work-related characteristics of the midwives, North Shoa Zone, Amhara Regional State, Ethiopia, 2025 (n = 473).

Variable Name	Category		Frequency	Percentage
Role of the Midwives	Clinician		453	95.77
	Clinician + Academician		19	4.02
	Facility Head		1	0.002
Facility Type	Health Center		271	57.29
	Primary Hospital		88	18.60
	General Hospital		79	16.70
	Referral Hospital		35	7.39
Employment Type	Permanent		451	95.34
	Temporary		22	4.65
Work Experience	1-5		177	37.42
	6-10		254	53.69
	> 10		42	8.87
Night Shift	Yes		402	84.98
	No		71	15.01
Number of Night Shifts	<3		219	46.30
	≥3		254	53.69
Working Hours	<40		112	23.67
	≥40		361	76.32
Working Unit	LD	Yes	380	80.34
		No	93	19.66
	ANC	Yes	215	45.45
		No	258	54.54
	GYN	Yes	70	14.79
		No	403	85.20
	FP	Yes	141	29.80
		No	332	70.19
Adjustable Couch	Yes		373	78.58
	No		100	21.14
Moving Heavy Objects	Yes		322	68.07
	No		151	31.92
Work in the same position for a long time	Yes		350	73.99
	No		123	26.00
Work in an awkward body posture	Yes		318	67.23
	No		155	32.76
See a large number of clients per day	Yes		391	82.66
	No		82	17.33
Adequate break each day	Yes		201	42.49
	No		272	57.50

### Magnitude of work-related musculoskeletal disorder

**Fig 2:**
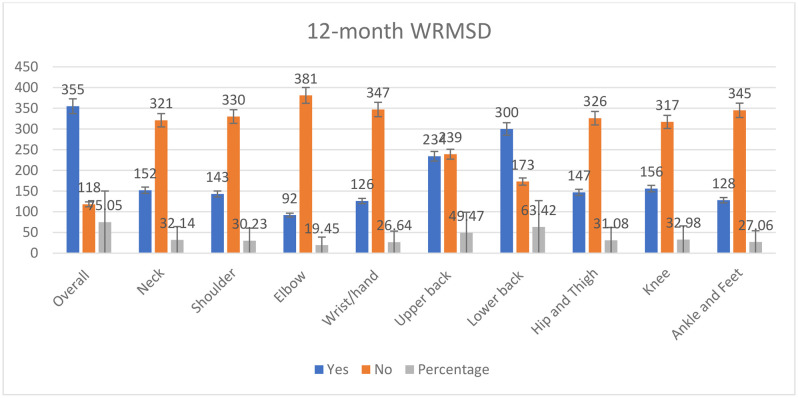
The prevalence of overall last 12-month WRMSD and the prevalence across the nine regions of midwives in the last 12 months, North Shoa Zone, Amhara Regional State, Ethiopia, 2025.

Three hundred fifty-five midwives reported pain in one of the nine body parts in the last 12 months, resulting in a 12-month overall WRMSD prevalence among midwives of 75.05% (95% CI: 71.15% − 78.95%). Regarding area-specific WRMSD, the lower back was the most affected axial body part, reported by 300 (63.4%, 95% CI: 59% − 68%) midwives. The upper back and neck were reported by 234 (49.47%, 95% CI: 44.97% − 53.98%) and 152 (32.14%, 95% CI: 27.93% − 36.34%) midwives, respectively. The knee and hip were the most affected appendicular body parts, reported by 156 (32.98%, 95% CI: 28.74% − 37.22%) and 147 (31.08%, 95% CI: 26.91% − 35.25%) midwives, respectively (Fig 2).

The effects of the condition range from limited activity to the need for medical assistance. About 45% (162) of the midwives with WRMSD were unable to perform their daily tasks while they had the condition, and 27% (96) of them sought treatment. From the 300 midwives who reported experiencing lower back pain, 117 (39%) indicated that their activity was limited, and 62 (20.67%) sought treatment; similarly, 74 (31.62%) and 50 (21.37%) of the midwives with upper back pain reported limitations in their activity and sought treatment, respectively (Table 3).

**Table 3:** Prevalence and Effect of Last 12 Months WRMSD on the Wellbeing of Midwives, North Shoa Zone, Amhara Regional State, Ethiopia, 2025.

Affected Body Part	12 Month WRMSD		Limited Activity			Seek Treatment		
	Yes (%)	No (%)	Yes	%	Valid %	Yes	%	Valid %
Neck	152(32.1)	321(67.9)	52	11.0	34.2	25	5.3	16.4
Shoulder	143(30.2)	330(69.8)	48	10.1	33.6	23	4.9	16.1
Elbow	92(19.5)	381(80.5)	37	7.8	39.4	19	4.0	20.2
Hand	126(26.6)	347(73.4)	40	8.5	31.5	23	4.9	18.1
Upper Back	234(49.5)	239(50.5)	74	15.6	31.6	50	10.6	21.4
Lower Back	300(63.6)	173(36.6)	117	24.7	39.0	62	13.1	20.7
Hip and thigh	147(31.1)	326(68.9)	46	9.7	31.3	28	5.9	19.0
Knee	156(33.0)	317(67.0)	49	10.4	31.2	28	5.9	17.8
Ankle	128(27.1)	345(72.9)	47	9.9	31.8	30	6.3	20.3

**Fig 3:**
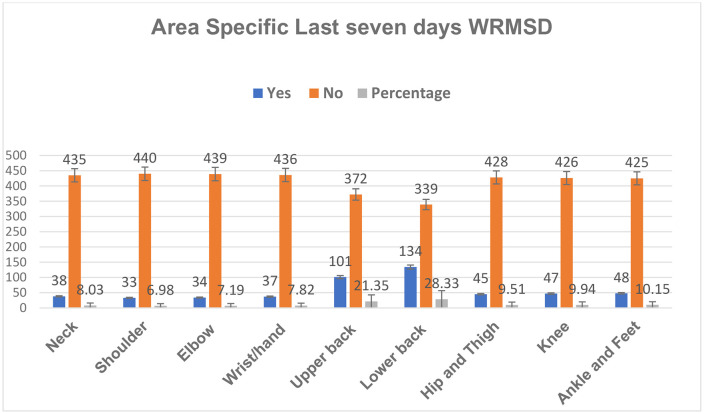
The prevalence of the last seven days WRMSD across the nine regions of the body among midwives, North Shoa Zone, Amhara Regional State, Ethiopia, 2025.

The prevalence of WRMSD in the last seven days was estimated for the nine body parts. The lower back was the most affected body part for the previous seven days of WRMSD, affecting 134 (28.33%, 95% CI: 24.27% − 32.39%) midwives. The upper back was the second most affected area, reported by 101 (21.35%, 95% CI: 17.66% − 25.05%). The least affected body part was the shoulder, reported by 33 (6.97%, 95% CI: 6.48% − 9.27%) midwives (Fig 3).

### Factors affecting the presence of WRMSD among midwives

Sex, education level, work experience, night duty, working unit, presence of an adjustable couch, working in awkward positions, maintaining one position for an extended period, seeing a high number of clients, and having adequate rest were included in the logistic regression model.

Working in awkward positions was retained in the backward elimination process as a significantly associated factor with the overall last 12-month WRMSD. Accordingly, midwives who reported working in awkward positions were 1.77 times more likely to experience pain in one of the nine assessed body parts compared to those who did not work in awkward positions.

When assessing associated factors for area-specific WRMSD of the axial body parts (neck, upper back, and lower back), midwives who reported working in the same position for extended periods were 1.81 and 1.73 times more likely to experience upper and lower back pain, respectively, compared to their counterparts. Similarly, midwives who saw a large number of patients each day were 1.69 and 1.85 times more likely to experience upper and lower back pain, respectively, than those who did not see a large number of clients in a day. Midwives who reported that their work involved moving heavy objects were 1.55 times more likely to experience upper back pain. Midwives who worked night shifts were 1.99 times more likely to experience lower back pain. Moreover, midwives who reported working in awkward positions were 2.82 times more likely to experience neck pain compared to those who did not report working in awkward positions (Table 4).

**Table 4:** Factors associated with overall last 12-month WRMSD, neck, upper back, and lower back of Midwives, North Shoa Zone, Amhara Regional State, Ethiopia, 2025.

The last 12-month overall WRMSD						
Variables		WRMSD		COR	AOR(95%CI)	P-Value
		Yes	No			
Week Work Hour	≤ 40 Hrs.	89	23	1	1	
	>40 Hrs.	266	95	0.72	0.58 (0.34–0.99)	0.048
Work in the same position for a long time	Yes	272	78	1.68	1.56 (0.98–2.51)	0.063
	No	83	40	1	1	
Awkward Position	Yes	250	68	1.75	1.77 (1.12–2.79)	0.014*
	No	105	50	1	1	
**12-month Neck WRMSD**						
Family size	≤2	63	172	1	1	
	3-4	62	119	2.45	2.04(1.10–3.78)	0.023*
	≥5	27	30	1.72	1.74(0.94–3.24)	0.080
Employment type	Permanent	150	301	4.98	4.66(1.05–20.67)	0.043*
	Temporary	2	20	1	1	
Awkward position	Yes	125	193	3.07	2.82 (1.74–4.56)	0.001*
	No	27	128	1	1	
**12-month Upper Back WRMSD**						
Moving heavy objects	Yes	173	149		1.55(1.04–2.32)	0.032*
	No	61	90	1		
Work in the same position for a long time	Yes	188	162		1.81(1.18–2.78)	0.006*
	No	46	77	1	1	
See large clients per day	Yes	204	187		1.69(1.02–2.79)	0.042*
	No	30	52	1	1	
**12-month lower back WRMSD**						
Education	Diploma	102	76	1	1	
	BSc	164	87	2.53	2.72(1.24–5.95)	0.012*
	MSc	34	10	1.80	1.91(0.88–4.12)	0.100
Night	Yes	265	137	1.99	2.22(1.31–3.76)	0.003*
	No	35	36	1	1	
Work in the same position for a long time	Yes	235	115	1.82	1.73(1.12–2.67)	0.013*
	No	65	58	1	1	
See large clients per day	Yes	258	133	1.85	1.85(1.12–3.05)	0.016*
	No	42	40	1	1	

* Significantly associated factors.

The regression analysis to identify factors associated with WRMSD of the appendicular part of the body (shoulder, elbow, wrist, hip, knee, and ankle) revealed that working in awkward positions was a common risk factor for all body parts. Accordingly, midwives who reported working in an awkward position are 2.01, 1.91, 2.41, 2.12, 1.74, and 2.83 times more likely to have shoulder, elbow, wrist, hip, knee, and ankle pain, respectively, compared with those who did not report working in an awkward position.

Midwives who reported seeing a large number of clients in a day are 2.18, 2.15, and 2.44 times more likely to have elbow, wrist, and ankle pain, respectively, compared to those who did not see a large number of clients. Similarly, permanently employed midwives are 5.65 and 4.09 times more likely to have elbow and ankle pain, respectively, compared to those who are temporarily employed.

Midwives with an MSc education level are 2.18 times more likely to have shoulder pain compared with those with a diploma. Midwives who see a large number of clients in a day are 3.39 times more likely to have elbow pain than those who did not see a large number of clients (Table 5).

**Table 5:** Factors associated with WRMSD of appendicular body regions (Shoulder, Elbow, Wrist/Hand, Hip/Thigh, Knee, Ankle/Feet) of midwives, North Shoa Zone, Amhara Regional State, Ethiopia, 2025.

12-month Shoulder WRMSD						
Variables		WRMSD		COR	AOR(95%CI)	P-Value
		Yes	No			
Education	Diploma	51	127	1	1	
	BSc	71	180	2.27	1.89(0.95–3.77)	0.070
	MSc	21	23	2.32	2.18(1.13–4.22)	0.020*
Awkward Position	Yes	111	207	2.06	2.01(1.27–3.20)	0.003*
	No	32	123	1	1	
**12 month Elbow WRMSD**						
Employment type	Permanent	91	360	5.25	5.65(0.74–43.25)	0.095
	Temporary	1	1			
Awkward position	Yes	74	244	2.31	1.91(1.08–3.38)	0.027*
	No	18	137	1	1	
See large clients per day	Yes	86	305	3.57	3.39(1.38–8.24)	0.007*
	No	6	76	1	1	
**12-month Wrist/Hand WRMSD**						
Employment type	Permanent	125	326	8.05	8.51(1.14–65.02)	0.039*
	Temporary	1	21	1	1	
Awkward position	Yes	103	215	2.75	2.41(1.44–4.04)	0.001*
	No	23	132	1	1	
See large clients per day	Yes	114	277	2.40	2.15(1.08–4.28)	0.028*
	No	12	70	1	1	
**12-month Hip/Thigh WRMSD**						
Marital Status	Single	41	129	1	1	
	Ever Married	106	197	1.69	1.46(0.94–2.26)	0.089
Awkward Position	Yes	116	202	2.29	2.12(1.33–3.360	0.002*
	No	31	124	1	1	
**12-Month Knee WRMSD**						
Awkward Position	Yes	121	197	2.12	1.89(1.21–2.98)	0.005*
	No	35	120	1	1	
See large clients per day	Yes	139	252	2.11	1.74(0.96–3.14)	0.067
	No	17	65	1	1	
**12-month Ankle and Feet WRMSD**						
Employment type	Permanent	126	325	3.88	4.09(0.92–18.32)	0.065
	Temporary	2	20	1	1	
Awkward position	Yes	107	211	3.24	2.83(1.66–4.81)	0.001*
	No	21	134	1	1	
See large clients per day	Yes	117	274	2.76	2.44(1.20–4.96)	0.014*
	No	11	71	1	1	

* Significantly associated factors.

The multicollinearity between predictor variables were assessed between all predictor variables, and there was no correlation between predictor variables with VIF value less than 5. The Hosmer-Lemeshow goodness-of-fit test suggested good fit of all the reported models with p-Value greater than 5.

## Discussion

This study was conducted to estimate the magnitude of WRMSD and identify its predictors among midwives. The magnitude of the overall last 12-month WRMSD, area-specific last 12-month WRMSD for the nine body regions, and seven days WRMSD of the nine body regions was assessed. In addition, associated factors with the overall last 12-month WRMSD and area-specific last 12-month WRMSD of the nine body regions were evaluated.

The prevalence of overall last 12-month WRMSD was estimated to be 75.05%; the most commonly affected body areas were the lower back, upper back, knee, and neck, affecting 63.42%, 49.5%, 33.0%, and 32.1% of the midwives, respectively. In addition, 39% of the midwives reported limited activity, and 20.67% sought treatment. Working in awkward positions was associated with the overall last 12-month WRMSD and the area-specific WRMSD for the neck, shoulder, elbow, wrist, hip, knee, and ankle. Seeing a large client was associated with the upper back, lower back, elbow, wrist, and ankle area WRMSD.

The prevalence of overall last 12-month WRMSD (75.05%) was similar to the study from Iran (77.5%) [9]. A study in Ghana among midwives and nurses reported that 75.3% of the participants reported having the condition [25]. A consistent finding was also seen among nurses in Ethiopia, where 73.8% of nurses working in public health facilities were affected [26]. However, it is lower compared with another study from Iran (96.7%), the UK (92%), and Tunisia (90.7%) [19,27,28]. This may be due to variations in the sociodemographics of the participants. The mean age of the participants was between 35 and 46 in the above studies, while the participants’ mean age in the current study was 29.43. In addition, the study participants were entirely female, while 46.7% of the participants in the current study were male. Age and sex were reported to have a significant effect on the development of WRMSD [6]. Two studies from Ghana and Iran reported lower prevalence of 53.8% and 67.6%, respectively. However, the sample size was 13 for Ghana and 50 for the Iran with similar mean age of 33 [8,16].

The prevalence of lower back (63.42%) and neck (32.14%) pain is lower compared with studies from Turkey, China, and the UK [18,19,29]. Similarly, the study participants were older and entirely female; the two factors have a significant effect [6]. A consistent finding was also seen among nurses in Ethiopia, where lower back and neck pain prevalence was 62.2% and 45.8% [26]. However, a study among nurses working in critical care units showed a very high prevalence of 97.6% WRMSD and 76% of lower back pain [30].

Such a high prevalence of WRMSD generally and lower back pain, with its effect on physical and functional limitation, may affect midwives’ quality of life as among nurses [31]. The poor quality of life may lead to occupational burnout, which in turn affects the quality of care [32]. This interwoven unfavorable condition may increase professionals’ need to leave their profession, which may create a vacuum in the health service sector [33,34].

Working in awkward positions was significantly associated with the overall last 12-month WRMSD, as well as the WRMSD related to the neck and all appendicular body parts (shoulder, elbow, wrist, hip, knee, and ankle) in the last 12 months. This finding is similar to those of studies from Ghana and Iran, which reported working in an awkward position as one of the perceived risk factors of WRMSD [25,27]. This may be due to working in a non-neutral position, which causes strain on muscles and can induce inflammation and tissue damage, leading to the development of WRMSD [4].

Working in the same position for longer was associated with upper and lower back area-specific WRMSD. This is similar to the study from China, which reported that long-lasting concentration significantly predicted WRMSD [18]. This may be due to fatigue and injury resulting from maintaining the same body posture for an extended period [4].

Seeing a large number of cases was associated with lower back, upper back, elbow, knee, and ankle area-specific WRMSD. This finding is similar to a study from China, which reported workload as an important predictor of WRMSD [18]. This may be due to seeing a large number of cases requiring repetitive movements that may cause tissue injury and induce inflammation that precipitates the development of WRMSD [2].

Moving heavy objects was significantly associated with the upper back area of WRMSD. This finding is similar to a study from Ghana [16]. This may be due to the forceful action in the occurrence of injury that will progress to WRMSD [2].

Permanently employed midwives are more likely to have neck, wrist, and ankle pain. This may be due to permanent staff being more likely to stay in a workplace and be exposed to occupational risks. Long-term exposure to occupational nuisances can lead to the development of WRMSD [4].

The condition has limited the activity of midwives and forced them to seek medical help. This finding is similar to studies from the UK and Ghana, which indicated that midwives sought assistance from GPs and physiotherapists [16,19]. This might be due to the severity, limiting nature, and chronic aspect of the condition, forcing midwives to seek help [35].

The primary strength of this study may be its generalizability since study participants were recruited from multiple institutions. Unlike most studies conducted among midwives, the study attempted to identify risk factors for general and area-specific WRMSD. However, this study did not involve other health professionals working in the maternity area and may not be generalized beyond midwifery professionals. The use of a cross-sectional study limited the ability of the study to provide causal inferences between health outcomes and the identified associated factors.

## Conclusion

Work-related musculoskeletal disorder is high among midwives, and it has affected the activity of a significant number of midwives. The lower back, upper back, knees, and neck are the areas most commonly affected. Working in an awkward position, seeing a large number of clients, and holding the same position for an extended period are common risk factors for the development of WRMSD.

WRMSD has limited the activity of midwives and forced them to seek help. WRMSD of the lower back was the most common cause of limited activity and seeking help.

Health facilities and authorities must devise a strategy to tackle WRMSD among midwives. Health facilities should train midwives to avoid working in awkward positions and to refrain from working in similar positions for extended periods. Furthermore, health facilities must adjust the professional-client ratio to the safest load level.

## Supporting information

S1 File S3 Data set for WRMSD among midwives. (XLSX)

## Abbreviation:

WRMSD: Work-related Musculoskeletal Disorder
NMQ: Nordic Musculoskeletal Questionnaire
CI: Confidence Interval;
COR: Crude Odds Ratio
AOR: Adjusted Odds Ratio
